# Supplementary material for: Glutamatergic cerebellar neurons differentially contribute to the acquisition of motor and social behaviors
Source: Nat Commun. 2023 May 15;14:2771. doi: 10.1038/s41467-023-38475-9 (PMC10185563; doi:10.1038/s41467-023-38475-9)
Supplement: Supplementary file 1 — Supplementary Information [file 41467_2023_38475_MOESM1_ESM.pdf]

Supplemental materials

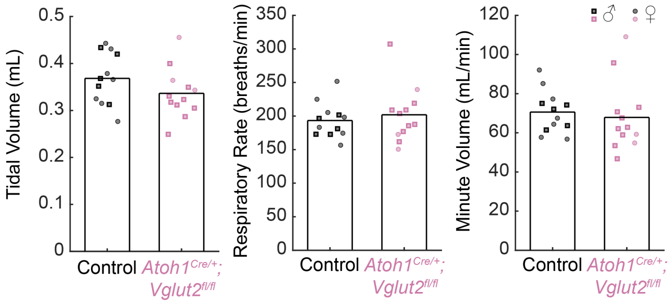

Supplemental Figure 1. Breathing control is not affected in adult *Atoh1*$^{Cre/+}$*;Vglut2*$^{fl/fl}$ mice. There are no differences detected in the Tidal Volume (p=0.163; d=0.576), Respiratory Rate (p=0.550, d=0.251), or Minute Volume (p=0.655, d=0.188) between control and *Atoh1*$^{Cre/+}$*;Vglut2*$^{fl/fl}$ mice. Control, total (female/male): N_{Female} = 5, N_{Male} = 7, N_{Total}=12; *Atoh1*$^{Cre/+}$*;Vglut2*$^{fl/fl}$: N_{Female} = 3, N_{Male} = 9, N_{Total}=12. Black circles and squares represent data points from control mice; reddish purple circles and square represent data points from *Atoh1*$^{Cre/+}$*;Vglut2*$^{fl/fl}$ mice. Squares represent data points from male mice, circles represent data points from female mice. Source data and detailed statistical results are available are provided as a Source Data file.

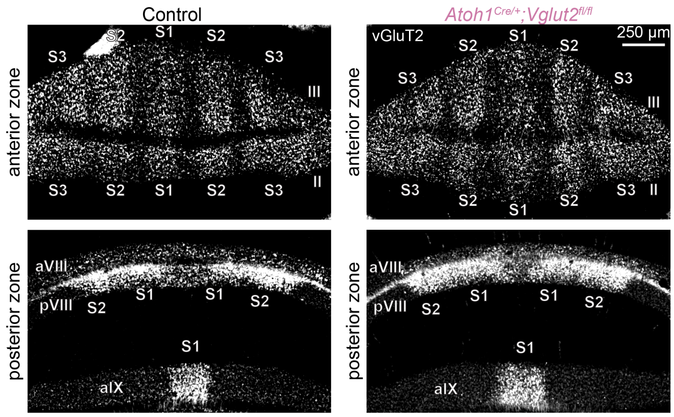

Supplemental Figure 2. VGluT2$^+$-expressing mossy fibers have the same stripe patterning in control and *Atoh1$^{Cre/+}$;Vglut2$^{fl/fl}$* mice. S1 = stripe 1, S2 = stripe 2, S3 = stripe 3. Roman numerals refer to the cerebellar lobules. The anterior (a) and poster (p) portions of lobules VIII and IX are shown**.** Images are representative of N=3 mice.

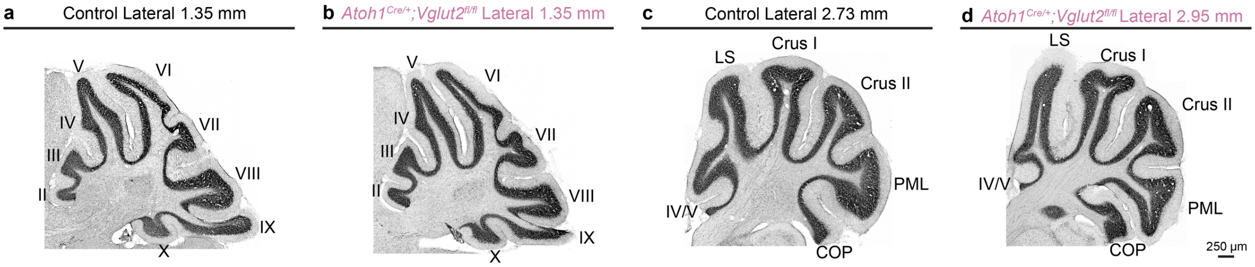

Supplemental Figure 3. Conditional deletion of *Vglut2* from the *Atoh1* lineage neurons does not lead to gross cerebellar morphological changes. Sagittal sections were stained with cresyl violet to visualize the cell nuclei and the gross architecture of the cerebellum. **a**. Medial section of an adult control mouse at a lateral coordinate of 1.35 mm. **b**. Medial section of an adult *Atoh1*$^{Cre/+}$*;Vglut2*$^{fl/fl}$ mouse at a lateral coordinate of 1.35 mm. **c**. Lateral section of an adult control mouse at a lateral coordinate of 2.73 mm. **d**. Lateral section of an adult *Atoh1*$^{Cre/+}$*;Vglut2*$^{fl/fl}$ mouse at a lateral coordinate of 2.95 mm. Purple represents DAPI fluorescence; green represents anti-Car8 (**a**), anti-Parvalbumin (**b**), anti-neurogranin (**c**), and anti-GFAP (**d**) immunofluorescence, respectively. Images are representative of multiple mice in each group. Control: N = 3; *Atoh1*$^{Cre/+}$*;Vglut2*$^{fl/fl}$: N = 4.

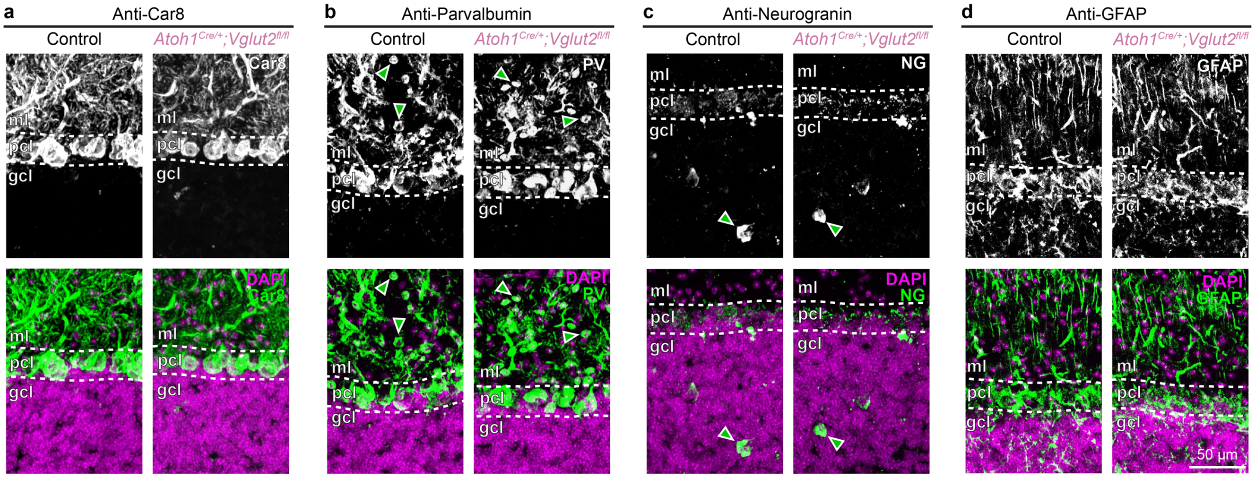

Supplemental Figure 4. Cerebellar interneurons were present and appeared unchanged in location and morphology in *Atoh1$^{Cre/+}$;Vglut2$^{fl/fl}$* mice. Dotted white lines indicate the borders of the Purkinje cell layer (pcl), which is located between the molecular layer (ml) and the granular cell layer (gcl). Scale = 50 µm. **a**. Carbonic anhydrase 8 (Car8) immunostaining marks the Purkinje cell and molecular layers as this marker strongly labels the somata and dendrites of Purkinje cells. **b**. Parvalbumin (PV) immunostaining labels Purkinje cells and molecular layer interneurons (green arrows). **c**. Neurogranin (NG) immunostaining marks the Golgi cells (green arrows). **d**. Glial Fibrillary Acidic Protein (GFAP) immunostaining shows the processes of Bergmann glia in the molecular layer. Images are representative of multiple mice in each group. Control: N = 3; *Atoh1$^{Cre/+}$;Vglut2$^{fl/fl}$*: N = 4.

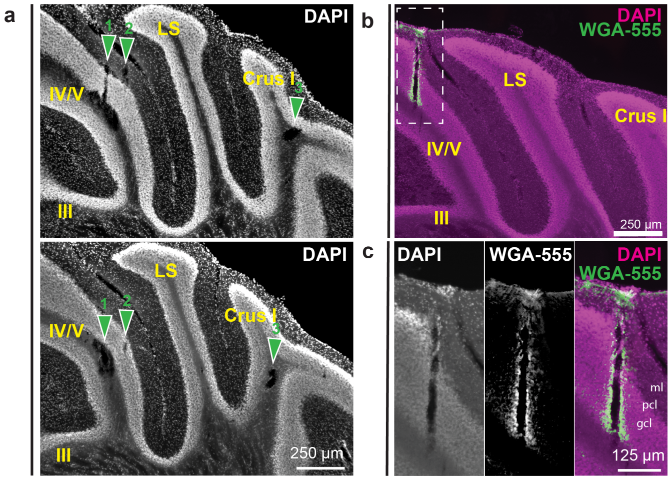

Supplemental Figure 5. Electrode tracks from electrophysiological recordings of Purkinje cells from different cerebellar regions. Coronal sections were stained for DAPI to visualize cell nuclei and gross architecture of the cerebellum while also revealing the electrode tracks in the tissue that were made during the recordings (a) and following a penetration with an electrode dipped in WGA-555 (b-c). **a.** Series of coronal sections depicting three electrode tracks created while recording from an adult *Atoh1*$^{Cre/+}$*;Vglut2*$^{fl/fl}$ mouse. Sections are spaced 80 µm apart with the top section taken from a more anterior position of the cerebellum (bregma = -5.88 mm). Each track is designated by an arrow and a number. Note that they include several cerebellar regions including lobules III, IV/V, and Crus I. **b.** Example of an electrode track created by an electrode dipped in WGA Alexa-555, demonstrating the shape of an electrode lesion in the brain. **c.** Magnified image of the electrode track shown in (b). Pcl = Purkinje cell layer, gcl = granule cell layer, ml = molecular layer, III = lobule III, IV/V = lobules IV/V, LS = lobule simplex, WGA Alexa-555 = wheat germ agglutinin conjugated to Alexa fluorophore-555 (for visual clarity the Alexa-555 is pseudocolored in green). Purple represents DAPI fluorescence; green represents WGA conjugated to a 555 fluorophore. Images are representative of N=3 mice.

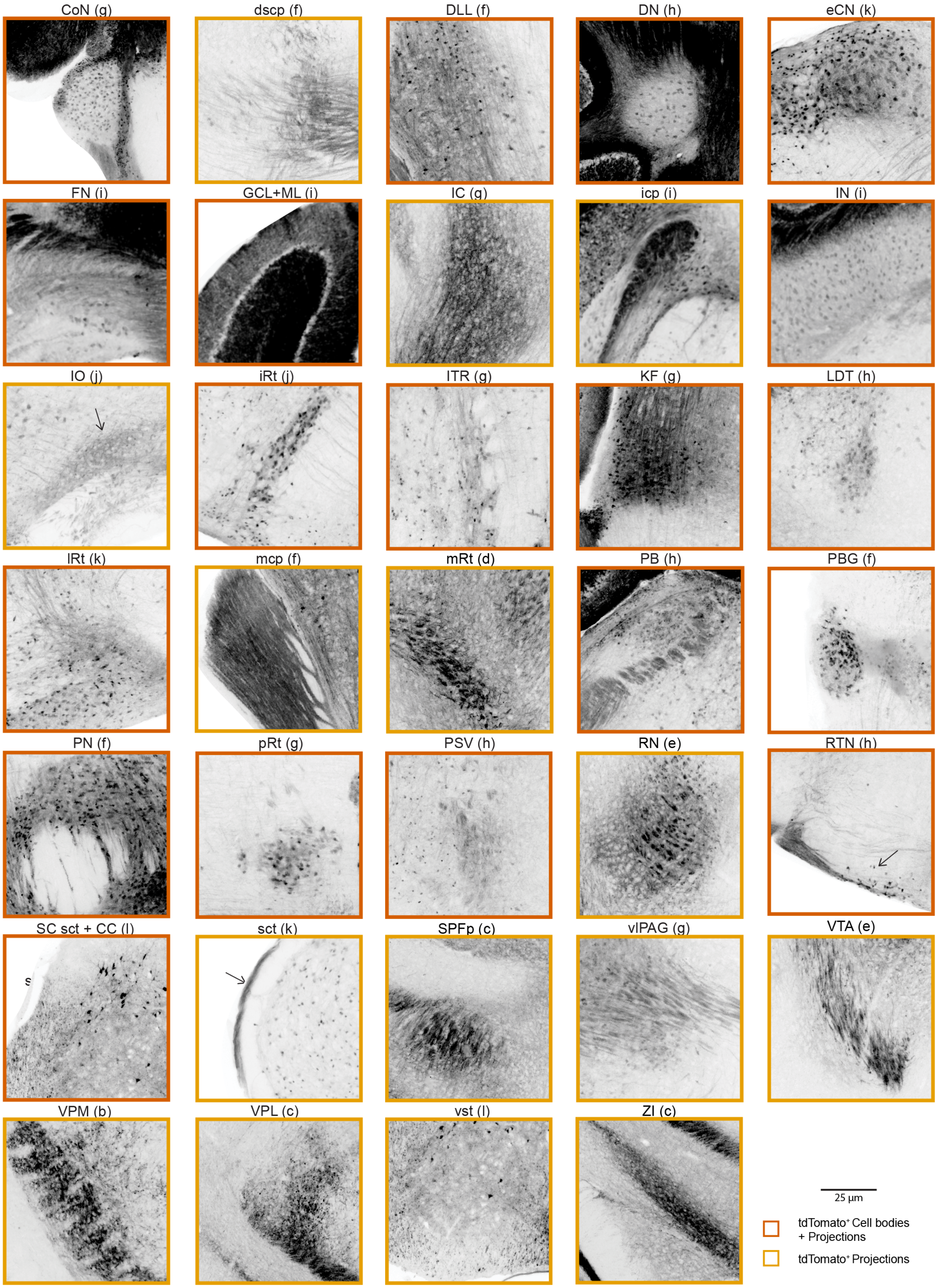

CoN (g)
dscp (f)
DLL (f)
DN (h)
eCN (k)
FN (i)
GCL+ML (i)
IC (g)
icp (i)
IN (i)
IO (j)
iRt (j)
ITR (g)
KF (g)
LDT (h)
lRt (k)
mcp (f)
mRt (d)
PB (h)
PBG (f)
PN (f)
pRt (g)
PSV (h)
RN (e)
RTN (h)
SC sct + CC (l)
sct (k)
SPFp (c)
vlPAG (g)
VTA (e)
VPM (b)
VPL (c)
vst (l)
ZI (c)
25 µm
tdTomato+ Cell bodies
+ Projections
tdTomato+ Projections

Supplemental Figure 6. High magnification view of the intersectional lineage labeling. Abbreviations: CoN = cochlear nucleus; dscp = decussation of dorsal superior cerebellar peduncle; DLL = dorsal lateral lemniscus; DN = dentate nucleus; eCN= external cuneate nucleus; FN = fastigial nucleus; GCL = granule cell layer; IC = inferior colliculus; icp = inferior cerebellar peduncle; IN = interposed nucleus; IO = inferior olive; iRt = intermediate reticular nucleus; ITR = intertrigeminal region; KF = Kölliker Fuse; LDT = lateral dorsal tegmental nucleus; lRt = lateral reticular nucleus; mcp = medial cerebellar peduncle; mRt = midbrain reticular nucleus; PB = parabrachial nucleus; PBG = parabigeminal nucleus; PN = pontine nuclei; pRt = pontine reticular nucleus; PSV = principal sensory nucleus of the trigeminal; RN = red nucleus; RTN = retrotrapezoid nucleus; sct = spinocerebellar tract; SPFp = subparafascicular nucleus, parvicellular part; vlPAG = ventral lateral periaqueductal gray; VPM = ventral posteromedial nucleus of the thalamus; VPL = ventral posterolateral nucleus of the thalamus; vst = ventral spinothalamic tract; VTA = ventral tegmental area; ZI = zona incerta. All images are shown at the same magnification. The light orange box means that only tdTomato$^+$ projections were observed, and the vermillion box means the cell bodies (and the projections) were observed. The letters in the brackets reference the coronal plane from which the higher magnification views were taken. The scale bar applies to all panels. Arrows point at the area of interest. Images are representative of N=3 mice.

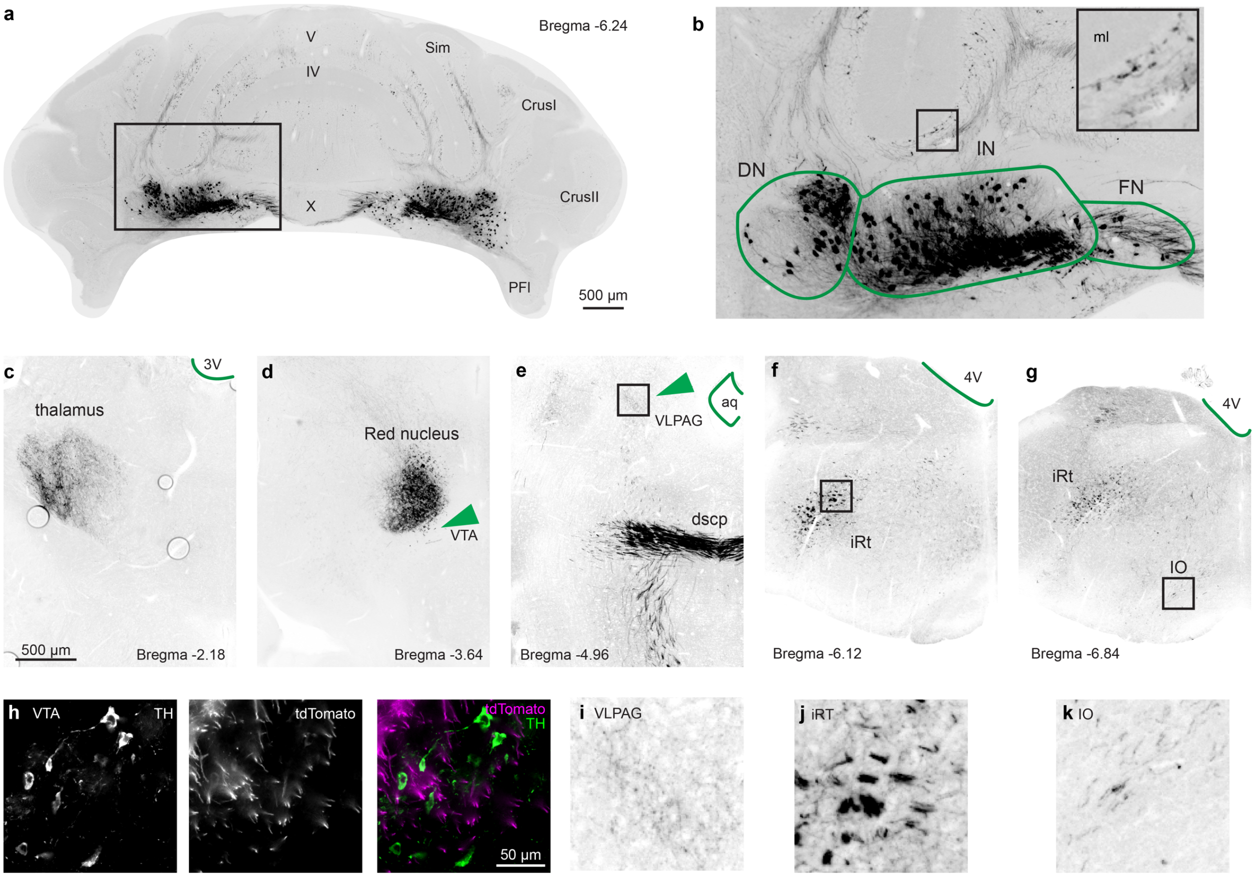

Supplemental Figure 7. Intersectional lineage labeling of *Ntsr1, Atoh1* neurons. tdTomato^{+} neuron labeling in *Atoh1$^{FlpO/+}$;Ntsr1$^{Cre/+}$;Rosa26$^{fsf\text{-}lsl\text{-}tdTomato}$* mice. **a.** Low magnification image of *Atoh1$^{FlpO/+}$;Ntsr1$^{Cre/+}$;Rosa26$^{fsf\text{-}lsl\text{-}tdTomato}$* cerebellum with nuclei cell labeling. **b.** Amplified magnification of box in **a.** Labeling of dentate nucleus (DN), interposed nucleus (IN), and fastigial nucleus (FN). Some nuclei project onto the molecular layer (ml) in the cerebellar cortex. **c.** Axon labeling in the thalamus. **d.** Axon labeling in the red nucleus and sparse axon labeling in the ventral tegmental area (VTA). **e.** Axon labeling in the decussation of the superior cerebellar peduncle (dscp) and sparse axon labeling in the ventral lateral periaqueductal gray (vlPAG). **f.** Axon labeling in the intermediate reticular nucleus (iRT). **g.** Axon labeling in the intermediate reticular nucleus (iRT) and sparse axon labeling in the inferior olive (IO). **h.** Confirmation of tdTomato^{+} axons traversing through TH^{+} neurons in the VTA. Purple shows tdTomato^{+} axons traversing, green shows anti-TH immunofluorescence. **i.** Higher magnification of box in **e. j.** Higher magnification of box in **f. k.** Higher magnification of box in **g.** Images are representative of N=3 mice.
