# Supplementary material for: Glutamatergic cerebellar neurons differentially contribute to the acquisition of motor and social behaviors
Source: Nat Commun. 2023 May 15;14:2771. doi: 10.1038/s41467-023-38475-9 (PMC10185563; doi:10.1038/s41467-023-38475-9)
Supplement: Supplementary file 3 — Reporting Summary [file 41467_2023_38475_MOESM3_ESM.pdf]

nature portfolio

Corresponding author(s): Roy V Sillitoe

Last updated by author(s): Sep 27, 2022

# Reporting Summary

Nature Portfolio wishes to improve the reproducibility of the work that we publish. This form provides structure for consistency and transparency in reporting. For further information on Nature Portfolio policies, see our Editorial Policies and the Editorial Policy Checklist.

## Statistics

For all statistical analyses, confirm that the following items are present in the figure legend, table legend, main text, or Methods section.

| n/a | Confirmed | |
|---|---|---|
| ☐ | ☒ | The exact sample size (*n*) for each experimental group/condition, given as a discrete number and unit of measurement |
| ☐ | ☒ | A statement on whether measurements were taken from distinct samples or whether the same sample was measured repeatedly |
| ☐ | ☒ | The statistical test(s) used AND whether they are one- or two-sided<br>*Only common tests should be described solely by name; describe more complex techniques in the Methods section.* |
| ☐ | ☒ | A description of all covariates tested |
| ☐ | ☒ | A description of any assumptions or corrections, such as tests of normality and adjustment for multiple comparisons |
| ☐ | ☒ | A full description of the statistical parameters including central tendency (e.g. means) or other basic estimates (e.g. regression coefficient) AND variation (e.g. standard deviation) or associated estimates of uncertainty (e.g. confidence intervals) |
| ☐ | ☒ | For null hypothesis testing, the test statistic (e.g. *F*, *t*, *r*) with confidence intervals, effect sizes, degrees of freedom and *P* value noted<br>*Give P values as exact values whenever suitable.* |
| ☒ | ☐ | For Bayesian analysis, information on the choice of priors and Markov chain Monte Carlo settings |
| ☒ | ☐ | For hierarchical and complex designs, identification of the appropriate level for tests and full reporting of outcomes |
| ☐ | ☒ | Estimates of effect sizes (e.g. Cohen's *d*, Pearson's *r*), indicating how they were calculated |

*Our web collection on statistics for biologists contains articles on many of the points above.*

## Software and code

Policy information about availability of computer code

| | |
|---|---|
| Data collection | Spike2 version7.09s |
| Data analysis | Spike2 version7.09s, MATLAB version R2021a, ImageJ 1.53e |

For manuscripts utilizing custom algorithms or software that are central to the research but not yet described in published literature, software must be made available to editors and reviewers. We strongly encourage code deposition in a community repository (e.g. GitHub). See the Nature Portfolio guidelines for submitting code & software for further information.

## Data

Policy information about availability of data

All manuscripts must include a data availability statement. This statement should provide the following information, where applicable:

- Accession codes, unique identifiers, or web links for publicly available datasets
- A description of any restrictions on data availability
- For clinical datasets or third party data, please ensure that the statement adheres to our policy

The datasets generated during and/or analysed during the current study are available in the Source Data file. Additional information is available from the corresponding author on reasonable request.

## Human research participants

Policy information about studies involving human research participants and Sex and Gender in Research.

| | |
|---|---|
| Reporting on sex and gender | NA |
| Population characteristics | NA |
| Recruitment | NA |
| Ethics oversight | NA |

Note that full information on the approval of the study protocol must also be provided in the manuscript.

# Field-specific reporting

Please select the one below that is the best fit for your research. If you are not sure, read the appropriate sections before making your selection.

☒ Life sciences ☐ Behavioural & social sciences ☐ Ecological, evolutionary & environmental sciences

For a reference copy of the document with all sections, see nature.com/documents/nr-reporting-summary-flat.pdf

# Life sciences study design

All studies must disclose on these points even when the disclosure is negative.

| | |
|---|---|
| Sample size | n cells, N animals. Sample size was determined based on previous experiments in our lab. |
| Data exclusions | No data was excluded. |
| Replication | All histology was replicated in minimally 3 animals and representative images were chosen for publication. |
| Randomization | NA - groups were determined based on mouse genotype. |
| Blinding | Mice were genotyped after conclusion of experiments to maintain blinding to genotype during the experiments. |

# Reporting for specific materials, systems and methods

We require information from authors about some types of materials, experimental systems and methods used in many studies. Here, indicate whether each material, system or method listed is relevant to your study. If you are not sure if a list item applies to your research, read the appropriate section before selecting a response.

### Materials & experimental systems

| n/a | Involved in the study | |
|---|---|---|
| ☐ | ☒ | Antibodies |
| ☒ | ☐ | Eukaryotic cell lines |
| ☒ | ☐ | Palaeontology and archaeology |
| ☐ | ☒ | Animals and other organisms |
| ☒ | ☐ | Clinical data |
| ☒ | ☐ | Dual use research of concern |

### Methods

| n/a | Involved in the study | |
|---|---|---|
| ☒ | ☐ | ChIP-seq |
| ☒ | ☐ | Flow cytometry |
| ☒ | ☐ | MRI-based neuroimaging |

## Antibodies

| | |
|---|---|
| Antibodies used | We used the following primary antibodies: guinea-pig (gp)-anti-VGluT2 (1:500; Synaptic Systems; #135404), rabbit (rb)-anti-VGluT1 (1:500; Synaptic Systems; #135302), rb-anti-Parvalbumin (PV) (1:1000; Swant; #PV 28), rb-anti-Neurogranin (NG) (1:500; Chemicon; #AB5620), rb-anti-carbonic anhydrase 8 (Car8) (1:500; Proteintech; #12391-1-AP), and sh-anti-tyrosine hydroxylase (TH) (1:500; Millipore; #AB1542). We used the following secondary antibodies which were conjugated to an Alexa-488 fluorophore: goat-anti-gp (1:1000; Invitrogen; #A11073), goat-anti-rb (1:1000; Invitrogen; # A32731), or goat-anti-sh (1:1000; Invitrogen; # A11015). |
| Validation | Conditional knockout, this and previous papers from our lab. We validated conditional knockout mice using IHC and IF experiments. To validate our primary antibodies, we include control staining in every experiment and have used null mice to test that our |

antibodies remain robust and specific to the intended antigen. Their expression is also consistent with that observed in our previous publications.

## Animals and other research organisms

Policy information about studies involving animals; ARRIVE guidelines recommended for reporting animal research, and Sex and Gender in Research

| | |
|---|---|
| Laboratory animals | We used the following mice for our experiments: Ai14(Rosalsl-TdTomato; JAX:007914), Ai65(Rosafsf-lsl-TdTomato; JAX:021875); Atoh1Cre;Atoh1FlpO(JAX:036541); Ntsr1Cre(MMRRC:030648),Vglut2IRES-Cre6(JAX: 028863); Vglut2fl (JAX:012898). (citations provided in text. All mice were kept under a 14 hr/10 hr light/dark cycle, daily temperature (68-72 F), humidity (30%-70%). Pup ages were indicated in the relevant figure panels and ranged between postnatal days 7-11. Adult mice were between two and fourteen months old. |
| Wild animals | No wild animals were used in the study. |
| Reporting on sex | We included both male and female mice in our study and reported them differently in the figures. We did not find any sex differences in any of our tests, therefore we combined male and female mice in all final statistical analyses. |
| Field-collected samples | No field collected samples were used in this study. |
| Ethics oversight | The Institutional Animal Care and Use Committee (IACUC) of Baylor College of Medicine (BCM) reviewed and approved all studies that involved mice. |

Note that full information on the approval of the study protocol must also be provided in the manuscript.
